# Protocol for Endoscopic Versus Open Cubital tunnel release (EVOCU): an open randomized controlled trial: EVOCU trial: Endoscopic Versus Open Cubital tunnel release

**DOI:** 10.1186/s12891-023-06234-y

**Published:** 2023-02-22

**Authors:** Philippe N. Sprangers, Egberta P. A. van der Heijden

**Affiliations:** 1 Department of Plastic, Reconstructive and Hand Surgery, Jeroen Bosch Ziekenhuis, PO Box 90153, 5200 ME ’s-Hertogenbosch, The Netherlands; 2 Department of Plastic, Reconstructive and Hand Surgery, Radboudumc, Nijmegen, The Netherlands

**Keywords:** Cubital tunnel, Elbow, Endoscopic, Open release, Ulnar nerve

## Abstract

**Background:**

Cubital tunnel syndrome is the second most common entrapment neuropathy of the upper extremity. Surgical decompression of the ulnar nerve aims to improve complaints and prevent permanent damage to the nerve. Open and endoscopic release of the cubital tunnel are both used in common practice, but none has proven to be superior. This study assesses patient reported outcome and experience measures (PROMs and PREMs respectively), in addition to objective outcomes of both techniques.

**Methods:**

A prospective single-center open randomized non-inferiority trial will take place at the Plastic Surgery Department in the Jeroen Bosch Hospital, the Netherlands. 160 patients with cubital tunnel syndrome will be included. Patients are allocated to endoscopic or open cubital tunnel release by randomization. The surgeon and patients are not blinded for treatment allocation. The follow-up time will take 18 months.

**Discussion:**

Currently, the choice for one of the methods is based on surgeon’s preferences and degree of familiarity with a particular technique. It is assumed that the open technique is easier, faster and cheaper. The endoscopic release, however, has better exposure of the nerve and reduces the chance of damaging the nerve and might decrease scar discomfort. PROMs and PREMs have proven potential to improve the quality of care. Better health care experiences are associated with better clinical outcome in self-reported post-surgical questionnaires. Combining subjective measures with objective outcomes, efficacy, patient treatment experience and safety profile could help differentiating between open and endoscopic cubital tunnel release. This could aid clinicians in evidence based choices towards the best surgical approach in patients with cubital tunnel syndrome.

**Trial registration:**

This study is registered prospectively with the Dutch Trial Registration under NL9556. Universal Trial Number (WHO-UTN) U1111-1267–3059. Registration date 26–06-2021. The URL: https://www.trialregister.nl/trial/9556

## Background

### Background and rationale

Cubital tunnel syndromeis the second most common entrapment neuropathy of the upper extremity [1, 2]. If conservative treatment fails to improve symptoms, surgery is indicated. Forsimple surgical decompression, two methods are being used in common practice: open release and endoscopic release. There is ongoing debate as to what constitutes the superior surgical approach [3–5].

Previous retrospective and prospective studies have compared the two surgical techniques in terms of clinical improvement, complications and patient satisfaction. Only three prospective studies [6–8] were performed of which only two [7, 8] were randomized, the number of participants was relatively small and they were of moderate quality. The American Society for Surgery of the Hand (2018) therefore states that research data on the optimal surgical treatment for cubital tunnel syndrome remains inconclusive [9]. A more large-sample, high-quality randomized controlled trial (RCT) is needed to verify the outcomes [6, 8, 10–13].

Moreover, according to recent overviews, PROMs and PREMs have enormous potential to improve the quality of care by increasing knowledge about people’s perspective on health quality [14, 15]. In current research on cubital tunnel release, however, PROMs and PREMs have not yet been included. Therefore, including these measurements in trials is an important addition to current literature. We intend to perform a more extensive open RCT with the required number of patients to have enough power to evaluate the clinical effect and patient experience with cubital tunnel release using validated objective outcome measures, including PROMs and PREM.

At the end of this study we hope to be able to make an evidence-based recommendation on which method has the best efficacy, patient treatment experience and safety profile. Moreover, this study will focus on the longitudinal validity of the Patient-Rated Ulnar Nerve Evaluation (PRUNE) in Dutch.

### Objectives

We hypothesize that endoscopic cubital tunnel release is more effective in treating cubital tunnel syndrome than open cubital tunnel release in both primary and secondary outcomes.

### Primary objective

To compare the change in PROMS between open and endoscopic cubital tunnel release using the Boston Carpal Tunnel Questionnaire (BCTQ).

### Secondary objectives

- To compare the change in PRUNE between open and endoscopic cubital tunnel release compared to the score of the BCTQ;
- To compare the PREM between open and endoscopic cubital tunnel release, and assess its association with PROM;
- To compare the post-operative recovery of sensibility;
- To compare the return to work/full function;
- To compare the complications;
- To compare the scar aesthetics;
- To compare the correlation between Visual Analog Scale (VAS) score (0-10, 0 being no pain to 10 being the worst pain), Bishop score (a 5 point scale, resulting in a total score of poor 0-2, fair 3-4, good 5-7, excellent 8-9) [16], two-point discrimination and both PROMS (BCTQ and PRUNE).

### Trial design

This is a prospective randomized non-inferiority trial with two parallel study groups to assess the efficacy, patient treatment experience and safety profile of the open or endoscopic release in patients with cubital tunnel syndrome.

## Methods

### Study setting

This single centre study will take place at the Plastic, Reconstructive and Hand Surgery Department of the Jeroen Bosch Hospital,’s-Hertogenbosch, the Netherlands.

### Eligibility criteria

#### Inclusion criteria

All eligible participants have to meet all of the following criteria:

-Complaints of idiopathic ulnar nerve entrapment at elbow, objectified clinically, with an electrophysiologic confirmed (EMG) diagnosis;
-Ability to measure the outcome of the study in this patient (e.g. life expectancy > 1 year, no planned relocation);
-Ability to speak and understand Dutch;
-Informed consent (written)

#### Exclusion criteria

A potential subject who meets any of the following criteria will be excluded:

- Age under 18;
- Not able to provide informed consent;
- Previous surgical cubital tunnel release or other surgery performed in the same elbow;
- Subluxation palpable during elbow flexion pre-operatively or occurring during surgery after release for which a transposition of the ulnar nerve is needed.

### Intervention

Patients will be randomized for the open cubital tunnel release or endoscopic cubital tunnel release. Both interventions are standard of care in the Jeroen Bosch Hospital. Patients who undergo a cubital tunnel release receive plexus or general anesthesia depending on the preference of the patient. After applying a pneumatic tourniquet, a longitudinal incision is made between the medial humeral epicondyle and the olecranon. The ulnar nerve is identified and released proximally and distally. The nerve branches of the flexor carpi ulnaris are identified. After wound closure with a resolvable suture, the patient receives a pressure bandage in a 45 degrees flexion and the bloodless field is abolished. Both the open and endoscopic release are performed in the same manner, except the length of the incision which is smaller in the endoscopic release; after identification of the ulnar nerve, the endoscope is inserted and the nerve is released with use of the endoscope. The HOPKINS ® Telescope 30° (4 mm, 18 cm) by Karl Storz. In addition, patients can watch the nerve release during the operation or after the surgery.

Both patient groups receive the same aftercare. After two days, the pressure bandage can be taken off and full active mobilization of the elbow is allowed. After two weeks full use of the arm (work, sport and axial load) is allowed. Patients can take acetaminophen postoperatively. We do not recommend physiotherapy.

### Allocation and blinding

#### Sequence generation, allocation and implementation

Patients will be assigned prospectively to one of the two treatment groups using a randomization module in Research Electronic Data Capture (REDcap) [17, 18]. The allocation sequence was made by the REDcap administrator. Randomization will not be stratified. The ratio will be 1:1.

Patients will be enrolled by EH. PS will assign participations to the intervention, according to the randomization module.

#### Blinding

Patients, doctors and researchers will not be blinded for the treatment allocation. The nature of our study does not allow for blinding of the aforementioned parties. Unblinding does therefore not apply.

### Outcomes

#### Primary outcome

The difference in change (Δ, preoperatively and postoperatively) in BCTQ score between both treatment groups at 3, 6 and 12 and 18 months follow-up.

#### Secondary outcomes

- The difference in change (Δ, preoperatively and postoperatively) in PRUNE score between both treatment groups at 3, 6, 12 and 18 months follow-up;
- The difference in PREM between both treatment groups at 3 months follow-up and its effect on the change (Δ, preoperatively and postoperatively) in PROM;
- The difference in post-operative recovery of sensibility between both treatment groups at 3, 6 and 12 months follow-up;
- The difference in time until return of full function (RTW) in days between both treatment groups;
- The difference amount of complications between both treatment groups during the follow-up period of 18 months;
- The difference in scar aesthetics between both treatment groups at 12 months follow-up;
- The (difference in) correlation between VAS score (0-10, 0 being no pain to 10 being the worst pain), Bishop score (a 5 point scale, resulting in a total score of poor 0-2, fair 3-4, good 5-7, excellent 8-9) [16], two-point discrimination and both PROMs (BCTQ and PRUNE) at 3, 12 and 18 months follow-up;
- The difference in surgical characteristics (duration of procedure, length of incision, amount of peroperative subluxation of the nerve after release) between both treatment groups.

#### Other parameters

In addition, disease characteristics (including affected side, duration of symptoms, the Tinel sign (positive or negative), handedness, use of splints, physiotherapy, avoidance of triggering factors and the McGowan classification) and demographic characteristics (including sex, age, type of work, smoking) will be collected. The McGowan classification is a three point scale assessing senbility and motor deficit: 1 Subjective symptoms, hypoesthesia; 2 Loss of sensibility, weakness of intrinsic musculature and or light muscle wasting and 3 Severe deficit of sensibility and motor functions.

### Follow-up

The follow-up takes 18 months post-operation. The schedule of trial enrolment is shown in Table 1. Study visits take place at 2 weeks, 3 months and 12 months post-operative with a surgeon or resident. These visits are standard of care. At 6 weeks follow-up, a phone consultation will be scheduled with a surgeon or resident. At 18 months patients will be asked to fill in questionnaires at home. These last two follow-up moments are additional to standard follow-up.

**Table 1:**
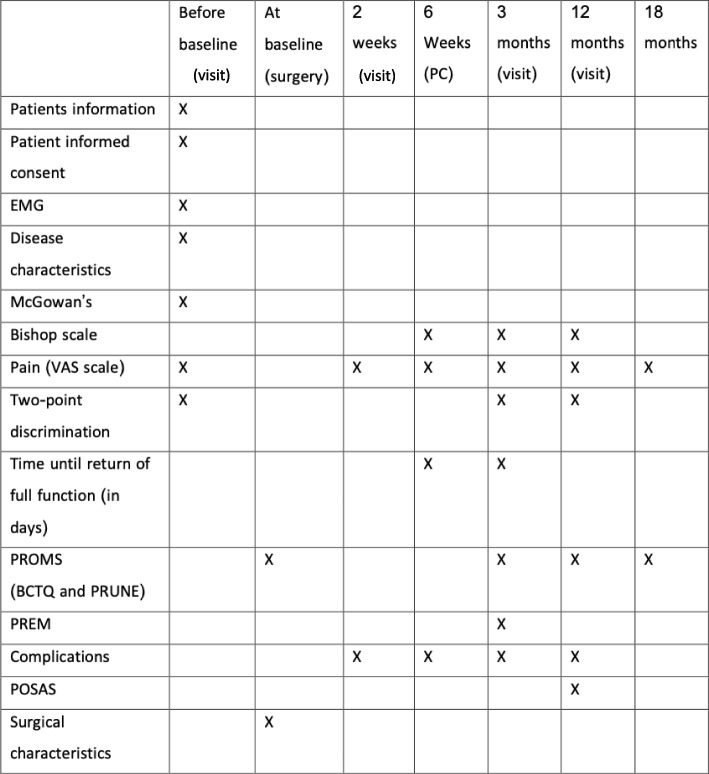
Trial Enrolment and Variables per Visit

### Data collection, data management, confidentiality and access to data

Data is collected from the electronic medical chart and the filled in questionnaire by patients. It is stored in a secure database, REDcap [17, 18]. All data stored will be stored anonymously. Patients will be identifiable through a unique code. This code will be stored at a secured server, only accessible by the principal investigator and one member of the research team collecting the data. The data will be stored for 15 years after the study has ended.

### Data monitoring, harms and auditing

A data monitoring committee will be provided for by the sponsor. They have no competing interest as the sponsor is a non-profit organization. No interim analyses will be performed. Serious Adverse Events and Adverse Events will be reported to the METC. Auditing will be performed by the METC.

### Sample size, recruitment and consent

Malay et al. identified a minimal clinically import change (MCIC) at 3, 6, and 12 months’ time-points of 0.4, 0.7, and 0.7 points on a 5-point scale for symptom severity score of the BCTQ questionnaire and 0.3, 0.3, and 0.4 points on a 5-point scale for function status scale [19]. The standard deviation used is 0.7, based on a 12 months follow-up after decompression [20].

Using a sample size formula for continuous outcomes in a longitudinal study, a total of 144 patients will be needed (α = 0.05, β = 0.80). Taking into account a dropout of 10%, we will include 160 patients.

Patients will be identified and invited for the study at the neurological outpatient clinic. Study information will be given and time to overthink participation. During the first outpatient clinic visit at the plastic surgery department, patients will get the chance to ask questions and sign written informed consent with the surgeon or resident present.

### Statistical methods

#### Primary study parameter

Difference in change in BCTQ: Linear generalized estimating equation (GEE) analyses will be used to study the longitudinal relationship between treatment group (determinant) on the one hand, and BCTQ score (outcome variable) on the other hand. This will be performed for the different domains of the BCTQ. This results in a single regression coefficient representing the population average difference in the outcome variable over time that incorporates between-subject and within-subject correlations.

#### Secondary study parameters

- Difference in change in PRUNE: Linear generalized estimating equation (GEE) analyses will be used to study the longitudinal relationship between treatment group (determinant) on the one hand, and BCTQ score (outcome variable) on the other hand. This will be done for the different domains of the PRUNE.
- Difference in PREM vs change in PROM: The PREM score is a continuous outcome and will be analyzed using an independent T-test if distributed normally and a Mann–Whitney U test when not. The change in PROM (BCTQ) score is a continuous outcome and will be analyzed using a paired T- test if distributed normally and a Wilcoxon signed rank test when not. To assess the association between PREM scores and PROM change scores, linear regression analyses will be performed. Multivariable regression models will be used to adjust for potential confounding of various patient and disease characteristics, including age, sex, body mass index, smoking and duration of disease. All domains of the PREM (pre-, intra- and postoperative care and communication) were introduced simultaneously in the same model as independent to determine to what extent the variation in treatment outcome between patients could be explained by the experience with health care, as measured by the PREM. The outcome is measured by the beta-coefficient, showing the change in PROM associated with 1 absolute point increase in PREM subscale.
- Sensibility: Sensibility is measured using two-point discrimination, resulting in a categorical outcome. The outcome includes > 2 categories and will be compared using a Chi-Squared test.
- RTW, Complications, Scar Aesthetics: Return of full function in days, number of complications and scar aesthetics are continuous outcomes and will be analyzed using an independent T-test.
- Correlation between VAS, Bishop, two-point discrimination and both PROMs: The association between VAS score, Bishop score, two-point discrimination and PROMs (both BCTQ and PRUNE) will be determined using the Pearson or Spearman correlation, depending on the data distribution. Analyses will be performed in two dimensions: 1) the correlation between preoperative VAS score, Bishop score, two-point discrimination and both PROMs and 2) the association between change in VAS score, Bishop score, two-point discrimination and both PROMs (12-month score minus preoperative score).

Difference in surgical characteristics: Characteristics of surgery includes continuous outcomes (operation time, length of the skin incision, length of decompression to distal in cm, length of decompression to proximal in cm and total length of decompression in cm) and a binary outcome (intraoperative nerve luxation). The continuous outcomes will be analyzed using an independent T-test and the binary outcome using Fisher exact testing.

#### Analysis plan

Descriptive statistics will be provided using mean with standard deviation (SD), median with interquartile range (IQR) or frequencies/percentages depending on the type of distribution of the data. Distribution of data will be assessed using histograms and skewing.

Values of *p* < 0.05 will considered statistically significant. All analysis will be on an intention- to-treat basis. We will correct for multiple testing, *P*-values will be corrected by the Bonferroni method [21].

For exclusion and dropout, numbers and reasons are reported to ensure internal validity. A flow- chart will show the number of patients lost during follow-up including reasons for the loss of follow-up. An analysis on basic characteristics will be performed to control for selection bias. Missing values will be imputed using multiple imputations when meeting the assumption of missing completely at random/missing at random (MCAR/MAR), as imputation will always increase precision and often also reduce bias [22, 23].

Table 1 shows the variables at each study visit.

## Discussion

As yet, no gold standard for cubital tunnel release exists, both the open and endoscopic approaches are commonly used. The two techniques have been compared, but not in an extensive RCT design with sufficient participants and including PROMs and PREMs.

Moreover, modern medicine increasingly focusses on improving treatment through understanding patient’s perspectives on health quality. PROMs and PREMs seem particularly important in surgical procedures as objectives measures might not adequately reflect success of these treatments. For that reason, PROMs and PREMs are increasingly used in clinical practice. Therefore, including these measurements in trials is an important addition to the current literature.

Randomized controlled trials (RCTs) are considered the most effective way to indicate relationships of cause and effect between interventions and outcomes.

It is expected that with this open randomized trial design including PROMs and PREMs, we will be able to make evidence based recommendations on best efficacy, patient treatment experience and safety profile regarding open and endoscopic cubital tunnel release. Moreover, since cubital tunnel is the second most common entrapment syndrome of the upper extremity, including patient’s experiences could increase quality of care and eventually quality of life on a large scale in the future.

## Data Availability

The dataset generated during this study are available from the corresponding author on reasonable request and will be published in a scientific article.

## Abbreviations

BCTQ: Boston Carpal Tunnel Questionnaire
EMG: Electromyography
EVOCU: Endoscopic versus Open Cubital Tunnel Release
GEE: Generalized Estimating Equation
IQR: Interquartile Range
MAR: Missing At Random
MCAR: Missing Completely At Random
MCIC: Minimal Clinically Important Change
METC: Medisch Ethische Toetsings Commissie
MREC: Medical Research Ethics Committee
PC: Phone Call
POSAS: The Patient and Observer Scar Assessment Scale
PREMs: Patient Reported Experience Measures
PROMs: Patient Reported Outcome Measures
PRUNE: Patient Rated Ulnar Nerve Evaluation
RCT: Randomized Controlled Trial
REDcap: Research Electronic Data Capture
RTW: Return To Work
SD: Standard Deviation
VAS: Visual Analog Scale
